# Effects of different modified atmosphere treatments on lipid oxidation in spiced beef at different storage temperatures

**DOI:** 10.1002/fsn3.2106

**Published:** 2021-01-23

**Authors:** Dan Hai, Xianqing Huang, Lianjun Song

**Affiliations:** ^1^ College of Food Science and Technology Henan Agricultural University Zhengzhou Henan China

**Keywords:** atmosphere packaging, lipid oxidation, spiced beef, storage temperature

## Abstract

The high moisture and nutrient contents of spiced beef make it popular with consumers but present challenges for its storage, as spoilage is a common phenomenon. Therefore, for identifying packaging methods to reduce spoilage during storage, this study investigated the effects of 5% O_2_ (low oxygen), 70% CO_2_ (high carbon dioxide), and 5% O_2_/70% CO_2_/25% N_2_ (compound group) on lipid oxidation in spiced beef in the test groups and a vacuum‐packed group (control) at storage temperatures of 4, 25, and 60°C. The pH, thiobarbituric acid (TBA), anisidine value (AV), and peroxide value (POV) of the spiced beef were determined. Results indicated that 70% CO_2_ and storage at 4 and 25°C showed the strongest ability to inhibit the rancidity in spiced beef. The 5% O_2_ group delayed both initial oxidation and secondary oxidation of lipids. Although the compound group significantly inhibited the rancidity in spiced beef at 60°C, it could not maintain such inhibition for long. Among all the groups, the 70% CO_2_ group demonstrated maximum inhibition of initial lipid oxidation and suppressed the secondary oxidation of lipids for the longest time. Thus, the modified atmosphere packaging with O_2_ and CO_2_ can regulate the fat oxidation in meat products and effectively improve their flavor maintenance.

## Graphical abstract

Spiced beef is a traditional Chinese cooked food product, and research on the mechanism of lipid oxidation of spiced beef in foreign countries is lacking. Atmosphere packaging technology was used for the first time for the preservation of traditional Chinese spiced beef. The effect of single gas component (5% O2/95% N2, 70% CO2/30% N2) on lipid oxidation of spiced beef was studied.

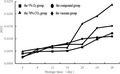

## 1 INTRODUCTION

Spiced beef is a traditional flavored meat product popular among many consumers in China (Huai‐Bin et al., 2008). Spiced beef is mostly sold in bulk, rich in protein and other nutrients, and has a high moisture content. Therefore, spiced beef is vulnerable to microbial growth even when stored at room temperature, leading to spoilage (Li et al., 2017). This restricts the production and circulation of spiced beef products (Jie‐Yun et al., 2010). Short shelf life and easily changed flavor are important factors that prevent spiced beef from being industrialized and hinder the development of a spiced beef industry of international standards. Lipid oxidation is responsible for the deterioration of the unique characteristic flavor of spiced beef that defines its essence (Xiong and Decker, 1995). Packaging provides protection against deterioration to many products (Yam et al., 2005); thus, different packaging methods can slow the lipid oxidation in spiced beef.

A wide range of domestic and foreign studies have been conducted on meat preservation technology. These studies reported that “atmosphere packaging” technology significantly improved the preservation of cold meat (Lizhen et al., 2004). Modified atmosphere packaging (MAP) refers to the removal and/or replacement of the atmosphere surrounding a product before sealing in a vapor barrier material (Mcmillin et al., 1998). All forms of MAP require the normal composition of atmospheric air to be removed or changed. MAP encompasses both aerobic packaging and anaerobic packaging of meat (Blakistone, 1998). MAP commonly uses gas comprising oxygen, nitrogen, and carbon dioxide. Nitrogen acts as a filler and does not change the physical and chemical properties of the sample. MAP is mostly used to control ripening and spoilage of fruits and vegetables (Prince and Brody, 1989). However, studies pertaining to the usefulness of MAP technology in spiced beef preservation are lacking.

To assess whether this packing method can improve the storage life of spiced beef, three types of atmosphere packaging were tested in this study: 5% O_2_/95% N_2_, 70% CO_2_/30% N_2_, and 5% O_2_/70% CO_2_/25% N_2_. The treated spiced beef was then stored at 4, 25, and 60°C, respectively. The lipid oxidation changes in spiced beef under low temperature and room temperature were studied under storage conditions of 4 and 25°C, respectively; further high temperature‐accelerated oxidation changes were studied at 60°C.

## 2 MATERIALS AND METHODS

### 2.1 Raw materials

Fresh beef was obtained as raw material (Henan Yisai Beef Co., Ltd, Jiaozuo, China), and the spiced beef was made using the patented method “*a method of making a sauced beef and a method for making beef sauce*.” The spiced beef was prepared in one simultaneous batch to ensure homogeneity of the beef sauce.

### 2.2 Preparation of spiced beef

The appropriate amount of beef was washed and placed in salt (approximately 6% of the beef weight) and pickled for three days. Simultaneously, a bag for seasoning spiced beef was prepared according to the patented method “*a method of making a sauced beef and a method for making beef sauce*” formula: 0.80% garlic, 1.10% ginger, 1.10% shallot, 1.40% crystal sugar, 0.11% clove, 0.23% amomum, 0.15% *Alpinia tonkinensis* Gagnep, 0.10% nutmeg, 0.15% Galangal Seed, 0.32% cinnamon, 0.26% pepper, 0.40% star anise, 0.10% licorice, 0.37% fennel, 0.08% vanilla, 0.26% codonopsis, 0.12% angelica, 0.30% tsaoko, 0.10% hawthorn, and 0.01% tangerine peel. The percentages were measured as a ratio of the raw beef total weight. After curing for three days, the seasoning bag was boiled for 10 min in water. The beef was then placed in the pot and boiled for 15 min. The heat was reduced, and the preparation was simmered for further 75 min. After the cooking time, the beef was cooled in the pan for 30 min, to obtain the finished spiced beef product.

### 2.3 Sampling and storage

The face of the spiced beef was removed, and the meat was sectioned into pieces of approximately 150 g that were then placed in the atmosphere packaging box and in a vacuum‐sealed bag. Samples were randomly divided into three groups, each containing 28 boxes, packed using an atmosphere packaging machine (Dajiang Machinery Equipment Co., Ltd, Wenzhou, China) and a vacuum packaging machine (Dajiang Machinery Equipment Co. Ltd), under the following atmosphere packaging conditions: inflatable time was 30 s, inflation pressure was 0.2 Pa, and atmosphere ratios were 5% O_2_/95% N_2_ (O_2_ group), 70% CO_2_/30% N_2_ (CO_2_ group), and 5% O_2_/70% CO_2_/25% N_2_ (compound group). The vacuum packaging conditions were as follows: vacuum time was 1.6 s and vacuum degree was 0.1 Pa. After each sample was packaged, it was immediately stored at a constant temperature of 4, 25, or 60°C. The samples stored at 4 and 25°C were measured on days 4, 8, 12, 16, 20, 24, and 28, whereas those stored at 60°C were measured on days 2, 4, 6, 8, 10, 12, and 14.

### 2.4 pH measurement

pH measurement was conducted according to China National Standard GB/T9695.5‐2008 “meat and meat products pH determination.” The pH meter (Thunder Magnetic PXSJ‐216 Meter, Shanghai, China) was calibrated before use. Three measurements at different locations were recorded for each sample (Li et al., 2017). pH of samples stored at 4 and 25°C was measured on days 4, 8, 12, 16, 20, 24, and 28, whereas that of those stored at 60°C was measured on days 2, 4, 6, 8, 10, 12, and 14.

### 2.5 Thiobarbituric acid value (TBA)

Using 10 g of beef as the sample, 50 mL of 7.5% trichloroacetic acid (containing 0.1% EDTA) was added to the sample and shaken for 30 min. Thereafter, the preparation was filtered twice with double filter paper, and 5 mL of the supernatant was treated with 5 mL of 0.02 mol/L TBA solution, incubated in a 95°C water bath for 50 min, removed and cooled to room temperature, and centrifuged for 5 min (1,600 rpm). The supernatant obtained was treated with 5 mL of chloroform. After stratification, the supernatant underwent colorimetric analysis at 532 and 600 nm (T6 Ultraviolet Spectrophotometer, Beijing, China). Absorbance was recorded, and a blank test was performed. The TBA value was calculated using equation 1 (Witte (20)):

(1) $${\text{TBARS = (A}}_{532}{\;\text{- A}}_{600}\text{)/155(1/m)}\times 72.6\times 100$$

where A_532_ = absorbance of the sample at 532 nm, A_600_ = absorbance of the sample at 600 nm, and M = quality of the sample (g).

### 2.6 Peroxide value (POV)

POV was determined according to the China National Standard GB5009.227‐2016. Briefly, an amount of spiced beef was added to the jar and treated with petroleum ether, the volume of which was 2 to 3 times greater than that of the sample. The sample was mixed well and allowed to stand for over 12 h. The sample was then treated with anhydrous sodium and subjected to funnel filtration. The filtrate was evaporated on a rotary evaporator (EYELA N‐1100 Rotary Evaporator, Japan) to obtain the oil sample. Next, 2–3 g of the oil sample was placed in a 250 mL iodine bottle and treated with 30 mL of a chloroform–glacial acetic acid mixture, following which 1 mL saturated potassium iodide solution was precisely added. The mixed sample was shaken for 0.5 min and placed in the dark for 3 min. Thereafter, the sample was collected, added to 100 mL of water, and immediately titrated to pale yellow using a standard solution of sodium thiosulphate (0.002 mol/L) and 1 mL of starch indicator. The titration was continued until the blue color disappeared; the blank group was set simultaneously. The POV is calculated using equation 2.

(2) $$\text{POV}=[\left(V‐V_{0}\right)\times N\times 0.1269]/m$$

where V = volume (mL) of thiosulphate standard solution consumed by the sample during titration (mL), V_0_ = volume (mL) of sodium thiosulphate standard solution consumed by the blank sample during titration solution volume, N = sodium thiosulphate standard titration solution concentration (mol/L), and m = quality of the sample (g)

### 2.7 Anisidine value (AV)

AVs were determined according to China National Standard GB/T24304‐2009 “animal and vegetable final anisidine determination.” The oil samples were prepared according to the method described in section 2.3 of the China National Standard GB/T24304‐2009. An amount of the sample was added to a 25‐mL volumetric flask, dissolved in 5 to 10 mL of isooctane, and diluted with isooctane to volume, to obtain the test solution. Next, 5 mL aliquots of the test solution were added to two scale test tubes, respectively. One test tube was treated with 1 mL of glacial acetic acid to produce the unreacted solution, whereas the other was treated with 1 mL of anisidine reagent to prepare the reaction solution; another test tube was treated with 5 mL of isooctane to produce a blank control solution. The unreacted solution, the reaction solution, and the blank solution were placed in the dark for 8 min at 23 ± 3°C. After storage, each solution was transferred to a clean cuvette within 2 min, and absorbances of the reaction solution, the unreacted solution, and the blank solution were individually measured at 350 nm. The total reaction time did not exceed 10 ± 1 min. The value of the anisidine in the sample was calculated using equation 3.

(3) $$\text{AV}=100\text{QV}/m\times 1.2\times (A_{1}‐A_{0}‐A_{2})$$

where V = volume (mL) of the solution sample; the unit is in milliliters; V = 25 mL, M = quality of the sample (g), and Q = concentration (g/mL) of the sample in the solution (according to the definition of AV); Q = 0.01 g/mL, A_0 =_ absorbance of the unreacted test solution, A_1 =_ absorbance of the reaction solution, and A_2 =_ absorbance of the blank solution.

### 2.8 Statistical analysis

The experiment was performed as a replicated test. Data were analyzed using one‐way analysis of variance using the SPSS 16.0 software (SPSS Inc., Chicago, IL, USA) for Windows. The results are expressed as mean (M) ± standard deviation (SD). Mean separation was performed via Duncan’s multiple range tests (*P <* 0.05).

## 3 RESULTS AND DISCUSSION

### 3.1 Effects of different modified atmosphere treatments on the pH of spiced beef at 4°C

pH value is the basic indicator of change in meat products (H. de Boer, 1992). The texture, juice, and color of food can be regulated by adjusting the pH value (Gault, 1985). The pH values of the 5% O_2_ group, the 70% CO_2_ group, and the compound group were 1.17%, 6.18%, and 3.17% higher than those in the vacuum group, respectively, on the 4th day of storage at 4°C (Table 1). On the 8th day of storage, the pH value of the 5% O_2_ group was 0.80% higher than that in the vacuum group, whereas the pH of the compound group was 1.77% higher than that in the vacuum group. There were no significant differences between the 70% CO_2_ group and the vacuum group. On the 12th day of storage, there was no significant difference between the 5% O_2_ group and the vacuum group. The pH of the 70% CO_2_ group was 4.17% higher than that in the vacuum group and 2.40% higher than that in the compound group. On the 16th day, there were no significant differences between the vacuum group and the 5% O_2_ and 70% CO_2_ groups. The pH value of the composite group was 1.71% lower than that in the vacuum group. The pH values of the 5% O_2_ group were 2.10%, 3.07%, and 1.29% higher than that in the vacuum group, the 70% CO_2_ group, and the compound group, respectively, on the 20th day. On the 24th day, there were no significant differences between the vacuum group and the 70% CO_2_ group. However, the pH of the 5% O_2_ group was 1.24% and 1.40% lower than that in the vacuum group and the compound group, respectively. However, there were no significant differences between the vacuum group, the 5% O_2_ group, the 70% CO_2_ group, and the compound group on the 28th day.

**TABLE 1:** Effects of different modified atmosphere treatment on the pH value of spiced beef at 4℃ storage temperatures

Storage days	4	8	12	16	20	24	28
Vacuum group	5.99 ± 0.02^d^	6.22 ± 0.02^c^	6.24 ± 0.01^b^	6.41 ± 0.03^ab^	6.19 ± 0.04^d^	6.43 ± 0.01^a^	6.10 ± 0.01^a^
5% O_2_ group	6.06 ± 0.01^c^	6.27 ± 0.02^b^	6.23 ± 0.01^b^	6.35 ± 0.06^bc^	6.32 ± 0.02^b^	6.35 ± 0.01^b^	6.29 ± 0.00^a^
70% CO_2_ group	6.36 ± 0.03^a^	6.22 ± 0.02^c^	6.50 ± 0.06^a^	6.44 ± 0.02^a^	6.38 ± 0.01^a^	6.44 ± 0.01^a^	6.32 ± 0.01^a^
Compound group	6.18 ± 0.02^b^	6.33 ± 0.03^a^	6.09 ± 0.01^c^	6.30 ± 0.02^c^	6.27 ± 0.03^c^	6.34 ± 0.02^b^	6.15 ± 0.02^a^

Note

Data within a column, showing different superscript letters, are statistically different (*p* < .05).

Compared with the pH of the vacuum group, the 5% O_2_ group, the 70% CO_2_ group, and the compound group delayed the rancidity in beef, and the 70% CO_2_ group showed the best effect, followed by the 5% O_2_ group. Although the compound group delayed the pH reduction effect initially, the pH reduction rate could not be maintained. Dan et al. (2014) studied changes in the pH of the compound group when stored at 10°C. The overall trend in pH change was essentially the same in this experiment. The up and down fluctuations of pH seen at later stages of storage may be because of the dissolution of CO_2_ on the surface of the meat, leading to formation of carbonic acid during the initial stages (Gill, 1996) and thus resulting in reduced pH values. An increase in microbial growth and reproduction causes protein oxidation, which results in increased pH during later storage (Mcmillin et al., 1998).

### 3.2 Effects of different modified atmosphere treatments on the TBA value of spiced beef at 4°C

Lipid oxidation in meat products is usually evaluated using the method for determination of 2‐thiobarbituric acid reactive substances (TBARS), which is widely used to evaluate lipid oxidation (Bornez et al., 2009). The TBA value of the 5% O_2_ group was higher than that in the vacuum group, when stored at 4°C (Table 2). This substantiates the conclusion of one study, which indicated that TBARS value increases with the oxygen concentration under low‐temperature storage conditions (Bao and Ertbjerg, 2015). The TBA value of the 70% CO_2_ group was slightly higher than that in the vacuum group. Although the initial TBA value of the compound group was smaller than that in the vacuum group, the growth rate in the compound group was higher than that in the vacuum group. The TBA values of the 5% O_2_ group and the 70% CO_2_ group were 0.06–0.17 and 0.01–0.14 higher than those in the vacuum group, respectively. The TBA values of the compound group were higher than that in the vacuum group, but the TBA values of the compound group were 0.17 higher than that in the vacuum group on the 28th day.

**TABLE 2:** Effects of different modified atmosphere treatment on the TBA value of spiced beef at 4℃ storage temperatures

Storage days	4	8	12	16	20	24	28
Vacuum group	0.13 ± 0.00^c^	0.13 ± 0.01^c^	0.15 ± 0.00^b^	0.15 ± 0.00^d^	0.16 ± 0.01^c^	0.16 ± 0.02^c^	0.20 ± 0.01^c^
5% O_2_ group	0.21 ± 0.01^a^	0.24 ± 0.01^a^	0.21 ± 0.01^a^	0.25 ± 0.01^a^	0.24 ± 0.01^a^	0.31 ± 0.01^a^	0.37 ± 0.02^a^
70% CO_2_ group	0.13 ± 0.01^c^	0.16 ± 0.01^b^	0.16 ± 0.00^b^	0.18 ± 0.00^c^	0.19 ± 0.01^b^	0.30 ± 0.01^a^	0.29 ± 0.02^b^
Compound group	0.15 ± 0.01^b^	0.17 ± 0.01^b^	0.22 ± 0.01^a^	0.22 ± 0.00^b^	0.18 ± 0.04^b^	0.20 ± 0.01^b^	0.37 ± 0.00^a^

Note

Data within a column, showing different superscript letters, are statistically different (*p* < .05).

Compared with the vacuum group, the TBA values of the 5% O_2_, the 70% CO_2_, and the compound group increased slightly, indicating that under storage conditions of 4°C, the slow lipid oxidation effect exerted by these three MAP samples was poor compared with that of the vacuum group. The 5% O_2_ group showed the worst effect, followed by the composite group. There was no significant difference between the 70% CO_2_ group and the vacuum group during initial storage, although the 70% CO_2_ group showed a slight increase during the later stages. The TBA values of the 5% O_2_ group and the compound group increased during the later stages of storage, whereas those of the vacuum group and the 70% CO_2_ group remained at a low level. This was probably because of the rapid propagation of aerobic bacteria, which promoted aerobic microbial decomposition of fat via oxidation. Oxygen may accelerate the increase in TBA values and promote lipid oxidation, as shown in Table 2 (Lund et al., 2007).

### 3.3 Effects of different modified atmosphere treatment on the AV of spiced beef at 4°C

AV represents the content of secondary oxidation products, such as a‐ and b‐alkanals and all compounds that react with p‐anisidine reagents (Guillen and Cabo, 2002). The AV of the 5% O_2_ group was lower than that in the vacuum group at 4°C, although it was slightly higher initially (Table 3). AV of the 70% CO_2_ group and the compound group was higher than that of the vacuum group. The initial AV of the compound group was larger, and its growth rate was higher, enabling it to finally exceed the AVs of other components.

**TABLE 3:** Effects of different modified atmosphere treatment on the anisidine value of spiced beef at 4℃ storage temperatures

Storage days	4	8	12	16	20	24	28
Vacuum group	0.40 ± 0.09^d^	0.81 ± 0.04^c^	1.15 ± 0.34^b^	1.59 ± 0.00^c^	1.85 ± 0.02^c^	2.14 ± 0.03^c^	2.62 ± 0.19^c^
5% O_2_ group	0.65 ± 0.40^c^	0.94 ± 0.06^b^	1.38 ± 0.14^b^	1.47 ± 0.77^b^	1.52 ± 0.44^c^	1.85 ± 0.05^d^	2.18 ± 0.03^d^
70% CO_2_ group	0.79 ± 0.65^b^	1.04 ± 0.06^b^	1.63 ± 0.01^a^	2.06 ± 0.12^a^	2.22 ± 0.11^b^	2.87 ± 0.17^b^	2.87 ± 0.28^b^
Compound group	1.21 ± 0.67^a^	1.18 ± 0.49^a^	1.63 ± 0.01^a^	2.16 ± 0.04^a^	2.73 ± 0.10^a^	3.22 ± 0.07^a^	3.48 ± 0.08^a^

Note

Data within a column, showing different superscript letters, are statistically different (*p* < .05).

The results showed that the 5% O_2_ group may have reduced the growth rate of AVs in the whole group. The delayed effects of the 70% CO_2_ group and the compound group were poor, the delayed effect of the compound group being the worst. Some studies have shown that the oxidation rate of oil during the induction period was lower than that in the oxidation period. The oxidation rate in later oxidation stages was higher than that in the early stage of oxidation. Furthermore, the oxidation rate was higher during later stages (Luan et al., 2009). The AV grew faster with time, consistent with the changes in AVs of the spiced beef.

### 3.4 Effects of different modified atmosphere treatments on the POV of spiced beef at 4°C

**FIGURE 1:**
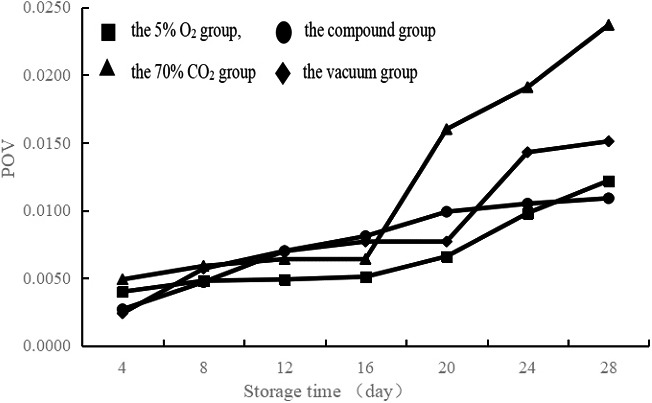
Effects of different modified atmosphere treatment (■: the 5% O_2_ group, ▲: the 70% CO_2_ group, ●: the compound group, ♦: the vacuum group) on the peroxidation value of spiced beef at 4℃ storage temperatures

POV is a common indicator of the early stages of oxidation in fats and oils (Mehta et al., 2015). The method of POV determination directly measures hydrogen peroxide concentrations by assessing the primary oxidation products resulting from the oxidation process (Cebi et al., 2017). The POV of the 5% O_2_ group was at a relatively low level compared with that of the vacuum group (Figure 1). The POV of the compound group was essentially the same as that of the vacuum group, but the POV of the compound group, although stable, was higher than that of the vacuum group at days 16 to 20 and lower than that of the vacuum group at days 20 to 24. The POV of the 70% CO_2_ group was lower than that of the vacuum group before day 16; however, it increased rapidly after day 16 and was much larger than the vacuum group.

Compared with the other treatment groups, the 5% O_2_ group inhibited lipid oxidation well and slowed the deterioration of spiced beef. Compared with the vacuum group, the compound group effectively inhibited lipid oxidation and maintained the inhibition of spiced beef well. The 70% CO_2_ group effectively inhibited lipid oxidation during the early stages, but this inhibitory effect was significantly reduced after 16 days.

### 3.5 Effects of different modified atmosphere treatments on the pH value of spiced beef at 25°C

Although there was no significant difference between the vacuum group, the 5% O_2_ group, and the compound group on the 4th day of storage at 25°C, the pH of the 70% CO_2_ group was 1.36% lower than that of the vacuum group (Table 4). On the 8th day of storage, the pH of the 5% O_2_ group, the 70% CO_2_ group, and the compound group was 1.39%, 5.39%, and 1.74% higher than that of the vacuum group, respectively. There were no significant differences between the vacuum group, the 5% O_2_ group, and the 70% CO_2_ group on the 12th day of storage, but the pH value of the compound group was 9.07% higher than that of the vacuum group. On the 16th, 20th, 24th, and 28th day of storage, the pH values of the 5% O_2_ group were 9.34%, 3.09%, 10.54%, and 9.01% higher than those of the vacuum group, whereas the 70% CO_2_ group were 6.06%, 6.53%, 5.54%, and 12.50% higher than those of the vacuum group. Simultaneously, pH values of the compound group were 1.39%, 6.53%, 26.79%, and 50.55% higher than those of the vacuum group.

**TABLE 4:** Effects of different modified atmosphere treatment on the pH value of spiced beef at 25℃ storage temperatures

Storage days	4	8	12	16	20	24	28
Vacuum group	5.89 ± 0.07^a^	5.75 ± 0.02^c^	5.51 ± 0.11^b^	5.78 ± 0.03^d^	5.51 ± 0.04^d^	5.60 ± 0.02^d^	5.44 ± 0.01^d^
5% O_2_ group	5.97 ± 0.01^a^	5.83 ± 0.04^b^	5.49 ± 0.05^b^	6.32 ± 0.02^a^	5.68 ± 0.05^c^	6.19 ± 0.03^b^	5.93 ± 0.03^c^
70% CO_2_ group	5.76 ± 0.03^b^	6.06 ± 0.04^a^	5.58 ± 0.04^b^	6.13 ± 0.02^b^	5.87 ± 0.02^b^	5.91 ± 0.03^c^	6.12 ± 0.02^b^
Compound group	5.87 ± 0.11^ab^	5.85 ± 0.02^b^	6.01 ± 0.01^a^	5.86 ± 0.01^c^	7.61 ± 0.02^a^	7.10 ± 0.05^a^	8.19 ± 0.02^a^

Note

Data within a column, showing different superscript letters, are statistically different (*p* < .05).

The pH value of the vacuum group had decreased to a low level at 28 days after storage, whereas the 5% O_2_, 70% CO_2,_ and compound groups delayed the rancidity in beef. The pH value of the 70% CO_2_ group was more stable and was maintained at approximately 5.6–6.1. Although the 5% O_2_ group delayed the reduction in pH, the overall trend was downward. The pH of the compound group decreased slightly during the first half of storage but increased rapidly during the second half of storage.

### 3.6 Effects of different modified atmosphere treatments on the TBA value of spiced beef at 25°C

The TBA values of the 5% O_2_ group, 70% CO_2_ group, and the compound group were higher than the vacuum group, when stored at 25°C (Table 5). The growth of the TBA value of the 70% CO_2_ group was slow and was almost equal to the vacuum group from the 12th day. The 5% O_2_ group and the compound group promoted the growth of the TBA value. The growth rate of the TBA value of the 5% O_2_ group was stable and eventually higher than 0.06 obtained from the vacuum group. The TBA value of the compound group increased rapidly after the 20th day and reached 0.43.

**TABLE 5:** Effects of different modified atmosphere treatment on the TBA value of spiced beef at 25℃ storage temperatures

Storage days	4	8	12	16	20	24	28
Vacuum group	0.09 ± 0.00^c^	0.12 ± 0.01^b^	0.15 ± 0.00^b^	0.16 ± 0.00^b^	0.15 ± 0.03^b^	0.16 ± 0.00^c^	0.18 ± 0.00^c^
5% O_2_ group	0.11 ± 0.00^b^	0.15 ± 0.01^a^	0.18 ± 0.00^a^	0.18 ± 0.00^a^	0.18 ± 0.00^a^	0.23 ± 0.01^b^	0.24 ± 0.01^b^
70% CO_2_ group	0.14 ± 0.00^a^	0.16 ± 0.00^a^	0.15 ± 0.01^b^	0.16 ± 0.00^b^	0.16 ± 0.00^b^	0.15 ± 0.01^c^	0.18 ± 0.01^c^
Compound group	0.15 ± 0.01^a^	0.15 ± 0.01^a^	0.17 ± 0.00^a^	0.18 ± 0.01^a^	0.18 ± 0.00^a^	0.32 ± 0.01^a^	0.43 ± 0.00^a^

Note

Data within a column, showing different superscript letters, are statistically different (*p* < .05).

The analysis demonstrated that although the initial TBA value of the 70% CO_2_ group was higher, its growth rate was slower than the other three packaging groups. This indicated that the 70% CO_2_ group could effectively delay the lipid oxidation in spiced beef. The delayed oxidative capacity of the 5% O_2_ group and the compound group was weaker than the vacuum group, where the delaying ability of the compound group was weaker than the 5% O_2_ group. Compared with the vacuum group, the delay lipid oxidation capacities of the 5% O_2_ group and the compound group were weak. With the prolongation of storage time, the delaying ability of the compound group was weaker than the 5% O_2_ group.

### 3.7 Effects of different modified atmosphere treatments on the AV of spiced beef at 25°C

At 25°C, the AVs of the 5% O_2_ group were low compared with the vacuum group, and the growth rate of the AVs remained low until the 28th day (Table 6). The AVs of the 70% CO_2_ group, which were lower than those of the vacuum group, increased gradually after 4 days and exceeded those of the vacuum control group, reaching a maximum on the 28th day. Compared with other treatment groups, the AV of the compound group grew fastest and remained at high levels.

**TABLE 6:** Effects of different modified atmosphere treatment on the anisidine value of spiced beef at 25℃ storage temperatures

Storage days	4	8	12	16	20	24	28
Vacuum group	0.19 ± 0.24^b^	0.20 ± 0.17^c^	0.53 ± 0.08^b^	0.56 ± 0.02^b^	0.73 ± 0.18^c^	0.83 ± 0.05^c^	1.64 ± 0.05^c^
5% O_2_ group	0.15 ± 0.04^d^	0.15 ± 0.17^d^	0.28 ± 0.00^c^	0.53 ± 0.40^c^	0.56 ± 0.00^d^	0.66 ± 0.20^d^	0.70 ± 0.01^d^
70% CO_2_ group	0.11 ± 0.05^c^	0.39 ± 0.47^b^	0.55 ± 0.07^b^	0.61 ± 0.13^b^	0.96 ± 0.03^b^	1.04 ± 0.32^b^	2.76 ± 0.21^a^
Compound group	0.67 ± 0.25^a^	1.05 ± 0.09^a^	1.42 ± 0.14^a^	1.53 ± 0.50^a^	2.10 ± 0.14^a^	2.12 ± 0.03^a^	2.59 ± 0.01^b^

Note

Data within a column, showing different superscript letters, are statistically different (*p* < .05).

Thus, compared with the vacuum group, the 5% O_2_ group took a long time to effectively delay the rise of the AV and suppressed the lipid oxidation in spiced beef. Compared with the vacuum group, the inhibitory effects exerted on lipid oxidation by the 70% CO_2_ group and the compound group were poor. Over time, the inhibitory effect of the 70% CO_2_ group became less significant, and the inhibitory effect of the compound group was worse than that in the other three groups.

### 3.8 Effects of different modified atmosphere treatments on the POV of spiced beef at 25°C

**FIGURE 2:**
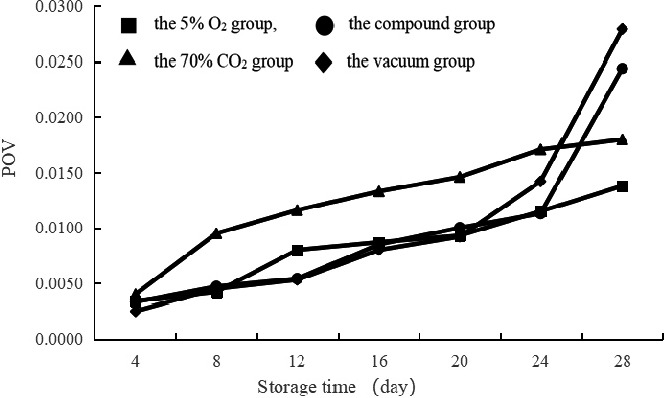
Effects of different modified atmosphere treatment (■: the 5% O_2_ group, ▲: the 70% CO_2_ group, ●: the compound group, ♦: the vacuum group) on the peroxidation value of spiced beef at 25℃ storage temperatures.

The POV of the vacuum group, the 5% O_2_ group, and the compound group displayed a similar trend during the first 20 days (Figure 2). The POV of the vacuum group was the largest, followed by the compound group and the O_2_ group. Although the POV of the 70% CO_2_ group was high, its final POV was lower than that of the vacuum group and the compound group.

The analysis demonstrated that the 5% O_2_ group had a greater ability to inhibit lipid oxidation in the spiced beef and that the oxidation level always remained at a low level. The inhibitory effects of the vacuum group and the compound group were similar and subsided after 24 days; however, the inhibitory effects of the vacuum group were largely reduced than those of the compound group. The ability of the 70% CO_2_ group to delay the lipid oxidation was weak, but the growth curve was gentler, indicating that the inhibitory effect could be maintained for a longer period.

### 3.9 Effects of different modified atmosphere treatments on the pH value of spiced beef at 60°C

There was no significant difference between the vacuum group, the 5% O_2_ group, 70% CO_2_ group, and compound group on the 2nd and 8th days of storage (Table 7). Further, there was no significant difference between the vacuum group and the 5% O_2_ group on the 4th day of storage. The pH values of the 70% CO_2_ group were 2.13% and 0.99% higher than those of the vacuum group and the compound group, respectively. On the 6th day of storage, the pH values of the 5% O_2_ group and the 70% CO_2_ group were 0.80% and 3.85% higher than those of the vacuum group, whereas pH of the compound group was 2.08% lower than that of the vacuum group. On the 10th day, there was no significant difference between the vacuum group and the compound group, and the pH values of the 70% CO_2_ group and 5% O_2_ group were 19.00% higher and 1.30% lower than those of the vacuum group, respectively. There was no significant difference between the vacuum group and the 5% O_2_ group on the 12th day, and the pH values of the 70% CO_2_ group and the compound group were 2.48% and 1.49% higher than the vacuum group, respectively. On the 14th day of storage, the pH values of the 5% O_2_ group, the 70% CO_2_ group, and the 5% O_2_ group were 1.96% lower, 1.47% lower, and 0.98% higher than those of the vacuum group, respectively.

**TABLE 7:** Effects of different modified atmosphere treatment on the pH value of spiced beef at 60℃ storage temperatures

Storage days	2	4	6	8	10	12	14
Vacuum group	6.22 ± 0.06^a^	6.09 ± 0.03^c^	6.23 ± 0.01^c^	6.17 ± 0.06^a^	6.16 ± 0.02^b^	6.04 ± 0.01^b^	6.11 ± 0.02^b^
5% O_2_ group	6.18 ± 0.04^a^	6.11 ± 0.02^bc^	6.28 ± 0.02^b^	6.17 ± 0.02^a^	6.08 ± 0.01^c^	5.99 ± 0.01^b^	5.99 ± 0.01^c^
70% CO_2_ group	6.22 ± 0.01^a^	6.22 ± 0.02^a^	6.47 ± 0.01^a^	6.17 ± 0.02^a^	7.33 ± 0.04^a^	6.19 ± 0.01^a^	6.02 ± 0.02^c^
Compound group	6.24 ± 0.05^a^	6.15 ± 0.02^b^	6.10 ± 0.04^d^	6.11 ± 0.16^a^	6.11 ± 0.05^bc^	6.13 ± 0.08^a^	6.17 ± 0.05^a^

Note

Data within a column, showing different superscript letters, are statistically different (*p* < .05).

The results indicated that the 5% O_2_ group, the 70% CO_2_ group, and the compound group exerted an inhibitory effect on the rancidity in spiced beef. The inhibitory effect of the 5% O_2_ group, which was higher than that of the vacuum group before the 8th day of storage, gradually decreases after the 8th day. The suppressive effect of the 70% CO_2_ group was more significant compared with the vacuum group and the 5% O_2_ group. Its inhibitory effect was significantly reduced after 8th day in a manner similar to the 5% O_2_ group. The inhibitory effect of the compound group was maintained at a relatively stable level during the 14 days of storage, and its inhibition of lipid oxidation was superior.

### 3.10 Effects of different modified atmosphere treatments on the TBA value of spiced beef at 60°C

The TBA values of the 5% O_2_ group were higher than those of the vacuum group at 60°C (Table 8). The TBA values of the 70% CO_2_ group, which were smaller relative to the vacuum group before the 8th day, increased after the 8th day, compared with the vacuum group. The TBA values of the compound group, which remained at a low level compared with the vacuum group before the 10th day, increased rapidly after 10th day. Analysis showed that before the 10th day, the lipid oxidation effect exerted by the compound group on spiced beef stored under 60°C was superior to that exerted by the vacuum group; however, this retardation effect decreased sharply after the 10th day. Although the 70% CO_2_ group delayed lipid oxidation in spiced beef more effectively during the first 8 days, this delaying effect gradually decreased in a manner similar to the vacuum group. Overall, TBA values of the 5% O_2_ group were lower than those of the other three treatment groups.

**TABLE 8:** Effects of different modified atmosphere treatment on the TBA value of spiced beef at 60℃ storage temperatures

Storage days	2	4	6	8	10	12	14
Vacuum group	0.15 ± 0.00^b^	0.18 ± 0.01^b^	0.18 ± 0.04^b^	0.20 ± 0.01^b^	0.23 ± 0.01^b^	0.23 ± 0.01^b^	0.24 ± 0.01^b^
5% O_2_ group	0.20 ± 0.00^a^	0.21 ± 0.00^a^	0.25 ± 0.01^a^	0.25 ± 0.01^a^	0.26 ± 0.00^a^	0.29 ± 0.03^a^	0.31 ± 0.00^a^
70% CO_2_ group	0.12 ± 0.00^c^	0.16 ± 0.01^c^	0.17 ± 0.00^b^	0.21 ± 0.00^b^	0.22 ± 0.01^b^	0.25 ± 0.01^b^	0.24 ± 0.03^b^
Compound group	0.13 ± 0.01^c^	0.13 ± 0.00^d^	0.14 ± 0.00^c^	0.15 ± 0.01^c^	0.16 ± 0.01^c^	0.26 ± 0.00^ab^	0.31 ± 0.01^a^

Note

Data within a column, showing different superscript letters, are statistically different (*p* < .05).

### 3.11 Effects of different modified atmosphere treatment on the AV of spiced beef at 60°C

The AVs of the 5% O_2_ group, 70% CO_2_ group, and the compound group were higher than the vacuum control group at 60°C (Table 9). The AVs of the 5% O_2_ group were slightly higher than the vacuum group. The growth rate of AV of the 70% CO_2_ group reduced with time. The AV of the compound group was the highest compared with the other three groups.

**TABLE 9:** Effects of different modified atmosphere treatment on the anisidine value of spiced beef at 60℃ storage temperatures

Storage days	2	4	6	8	10	12	14
Vacuum group	0.21 ± 0.06^c^	0.28 ± 0.12^c^	2.04 ± 0.18^b^	2.47 ± 0.18^bc^	2.85 ± 0.08^d^	3.26 ± 0.40^c^	5.36 ± 0.16^c^
5% O_2_ group	0.23 ± 0.05^c^	0.83 ± 0.34^b^	2.35 ± 0.10^a^	2.79 ± 0.49^a^	4.08 ± 0.06^a^	4.25 ± 0.10^b^	6.01 ± 0.18^b^
70% CO_2_ group	0.37 ± 0.08^b^	1.77 ± 0.10^a^	2.40 ± 0.05^a^	2.49 ± 0.00^c^	3.35 ± 0.08^c^	4.05 ± 0.20^b^	4.79 ± 0.19^d^
Compound group	0.44 ± 0.20^a^	0.99 ± 0.09^b^	1.99 ± 0.53^a^	2.51 ± 0.38^b^	3.75 ± 0.31^b^	6.19 ± 0.40^a^	7.35 ± 0.20^a^

Note

Data within a column, showing different superscript letters, are statistically different (*p* < .05).

Compared with the vacuum group, the inhibition of lipid oxidation in the 5% O_2_ group, the 70% CO_2_ group, and the compound group was poor at 60°C, and these could not effectively inhibit the lipid oxidation in the beef. Although the 70% CO_2_ group did not inhibit lipid oxidation well during the first 12 days, as compared with the vacuum control group, the AVs of the 70% CO_2_ group were lower than the vacuum group after the 12th day. This indicated that the 70% CO_2_ group was better at long‐term inhibition of lipid oxidation and that its ability to continue inhibiting lipid oxidation was relatively strong. The inhibitory effect of the 5% O_2_ group was slightly worse than the vacuum group, whereas the inhibitory effect of the compound group was the worst.

### 3.12 Effects of different modified atmosphere treatments on the POV of spiced beef at 60°C

**FIGURE 3:**
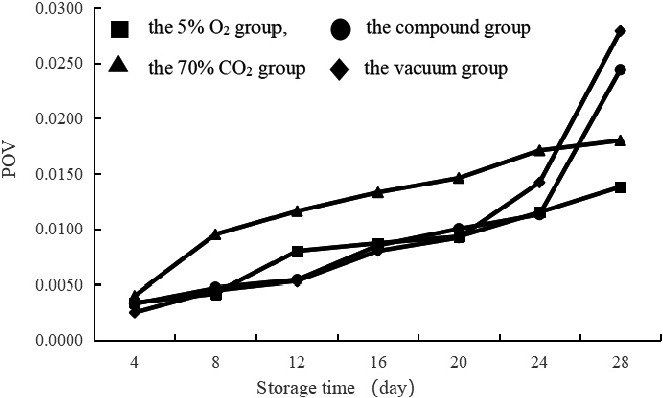
Effects of different modified atmosphere treatment (■: the 5% O_2_ group, ▲: the 70% CO_2_ group, ●: the compound group, ♦: the vacuum group) on the peroxidation value of spiced beef at 60℃ storage temperatures.

The peroxide curves of the 5% O_2_ group, the 70% CO_2_ group, and the compound group were higher than the vacuum group until 10 days of storage (Figure 3). The POVs of the 70% CO_2_ group were less than the 5% O_2_ group and the compound group, and the peroxide curves of the 5% O_2_ group and the compound group were approximately similar. After 10 days of storage, the POVs of the vacuum group increased rapidly and exceeded those of the other three groups. The POVs of the 5% O_2_ group were higher than the 70% CO_2_ group and compound group during 14 days of storage.

The results showed that the degrees of inhibition of lipid oxidation by the 5% O_2_ group, the 70% CO_2_ group, and the compound group were weaker than the vacuum group before 10 days of storage, indicating that the 5% O_2_ group, the 70% CO_2_ group, and the compound group could not delay the deterioration of spiced beef effectively. The inhibitory effect of the vacuum group was significantly decreased after 10 days, whereas the inhibitory effect of the 5% O_2_ group was weaker than the 70% CO_2_ group, likely because of exposure to high temperatures for a long time. In a pure oxygen environment, the 5% O_2_ group was more effective at promoting lipid oxidation, which led to an increase in the POV.

## 4 CONCLUSIONS

In this study, experimental data showed that when storing at 4°C, the 70% CO_2_ group slowed down changes in the pH value and effectively retarded the rancidity in spiced beef. The TBA value of the 70% CO_2_ group and vacuum group increased slowly and may have led to effective inhibition of fat oxidation, although the inhibitory effect exerted by the 70% CO_2_ group during later storage was poor. The 5% O_2_ group delayed the increase in AVs and POVs and inhibited both early oxidation and secondary lipid oxidation. Furthermore, at 25°C, the 70% CO_2_ group slowed down the change in pH value and effectively retarded the rancidity in spiced beef. The TBA values of the 70% CO_2_ group increased more slowly as the vacuum group. However, the inhibition effect under the 25°C storage condition was slightly stronger than that at 4°C. Thus, the 5% O_2_ group was best at inhibiting both initial oxidation and secondary oxidation of lipids. At 60°C, the pH of the compound group showed a slow constant decrease, which effectively delayed the rancidity in the beef. Moreover, the delaying effect of the 70% CO_2_ group decreased rapidly at day 14. In the first 10 days, the TBA of the compound group was increased slowly and better inhibited lipid oxidation, However, the suppression effect of the 70% CO_2_ group was better for 12–14 days. The AVs of the 70% CO_2_ group were higher during the early stages, but the growth rate was low, and the AVs of the 70% CO_2_ group were lower than those of the vacuum group at later stages. This indicated that the initial effect exerted by the 70% CO_2_ group on secondary oxidation was average but produced a prolonged inhibitory effect. Compared with other groups, the POVs of the 70% CO_2_ group grew slowly and remained at a low level, indicating that the 70% CO_2_ group effectively inhibited the initial oxidation of lipids.

This study investigated the effects of different modified atmosphere treatments on lipid oxidation in spiced beef at different storage temperatures. It provides data that support the undertaking of an in‐depth study of the mechanisms underlying lipid oxidation in spiced beef. Furthermore, it provides theoretical support for the industrialization of spiced beef production.

## CONFLICT OF INTEREST

The authors declare that they have no conflict of interest.
